# *PNKP* Mutations Identified by Whole-Exome Sequencing in a Norwegian Patient with Sporadic Ataxia and Edema

**DOI:** 10.1007/s12311-016-0784-y

**Published:** 2016-05-10

**Authors:** C. Tzoulis, Paweł Sztromwasser, Stefan Johansson, Ivar Otto Gjerde, Per Knappskog, L. A. Bindoff

**Affiliations:** 1 Department of Neurology, Haukeland University Hospital, 5021 Bergen, Norway; 2 Department of Clinical Medicine, University of Bergen, Bergen, Norway; 3 K.G. Jebsen Centre for Neuropsychiatric Disorders, Department of Clinical Science, University of Bergen, Bergen, Norway; 4 Center for Medical Genetics and Molecular Medicine, Haukeland University Hospital, Bergen, Norway; 5 Computational Biology Unit, Department of Informatics, University of Bergen, Bergen, Norway

**Keywords:** Ataxia, Oculomotor apraxia, Edema, Hypoalbuminemia, AOA4

## Abstract

We identified PNKP mutations in a Norwegian woman with AOA. This patient had the typical findings with cognitive dysfunction, peripheral neuropathy, cerebellar dysarthria, horizontal nystagmus, oculomotor apraxia, and severe truncal and appendicular ataxia. In addition, she had hypoalbuminemia and massive lower limb edema which showed some improvement with treatment. Exome sequencing identified two heterozygous mutations, one in exon 14 (c.1196T>C, p.Leu399Pro) and one in exon 16 (c.1393_1396del, p.Glu465*). This is the first non-Portuguese patient with AOA due to PNKP mutations and provides independent verification that PNKP mutations cause AOA.

## Introduction

Ataxia with oculomotor apraxia (AOA) is an autosomal recessive disorder characterized by the combination of progressive spinocerebellar ataxia, sensory and motor peripheral neuropathy, and various extrapyramidal features including choreoathetosis, dystonia, and tremor.

Four genetically distinct types have been described. AOA1 (MIM#208920) caused by mutations in *APTX* encoding aprataxin is characterized by hypoalbuminemia and hypercholesterolemia [1]. AOA2 (MIM#606002) due to mutations in the *SETX* gene encoding senataxin commonly shows increased serum alpha-fetoprotein levels [2]. AOA3 (MIM#615217) is caused by PIK3R5 encoding phosphoinositide-3-kinase, regulatory subunit 5 [3]. A fourth type termed AOA4 (MIM#616267) was described recently in eight Portuguese families carrying mutations in the *PNKP* gene encoding polynucleotide kinase 30-phosphatase, a protein involved in DNA-damage repair [4]. AOA4 starts in early childhood and is clinically characterized by dystonia, ataxia, oculomotor apraxia, sensorimotor peripheral neuropathy with distal amyotrophy, and cognitive impairment. Hypoalbuminemia, hypercholesterolemia, and elevated alpha-fetoprotein have been described in some patients but neither occurs consistently. The clinical and genetic features of AOA are summarized in the table.

We have identified the first non-Portuguese patient with AOA due to *PNKP* mutations. Our findings confirm the role of *PNKP* in causing AOA an independent population.

## Case Report

### Clinical Description

The patient, a 50-year-old woman of Norwegian ancestry without family history of neurological disease, was referred to our center due to progressive gait unsteadiness. She was born to non-consanguineous parents after a normal pregnancy, delivery, and early psychomotor development. At age 6, she developed progressive gait unsteadiness and general motor clumsiness. From her early twenties, she complained of numbness and reduced sensation with a glove and stocking distribution and distal weakness in the lower limbs. At the age of 30 years, she became wheel-chair-bound.

**Fig. 1:**
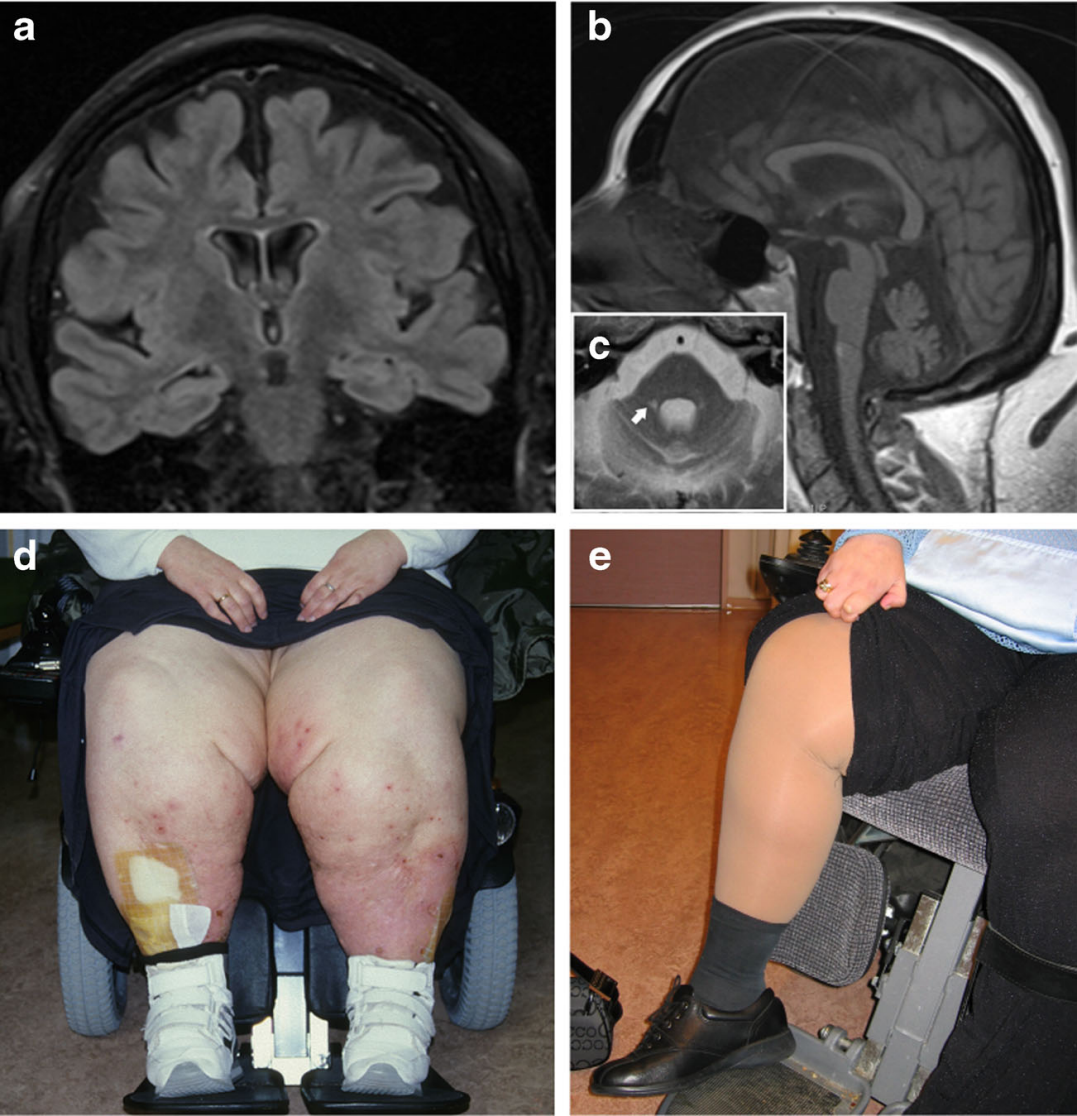
Clinical and radiological features of our patient. **a** Coronal FLAIR-T2 MRI showing generalized cerebral atrophy and ex vacuo ventricular dilation. **b** Sagittal T1-weighted MRI showing midline cerebellar atrophy. **c** Axial T2-weighted MRI showing cerebellar and pontine atrophy with dilation of the fourth ventricle and punctate bilateral high signal lesions at the base of the middle cerebellar peduncles (*arrow*). **d** Photograph of the patient showing severe bilateral edema of the lower limbs. **e** Improvement of the edema after albumin infusion and compression treatment

Clinical examination at the age of 31 revealed moderate cognitive dysfunction. She had cerebellar dysarthria, horizontal nystagmus, oculomotor apraxia, and severe truncal and appendicular ataxia with dysmetria and dysdiadochokinesia. She could not stand or walk unsupported. There was flaccid paralysis at the ankles, bilateral *pes cavus* and hammer toe deformities, and global hypoesthesia in the distal limbs. She had generalized subcutaneous edema (anasarca), which was most pronounced in the lower limbs (Fig. 1d).

Nerve conduction velocities (NCVs) and electromyography (EMG) were technically difficult in the lower limbs due to the edema. In the upper limbs, there was decreased conduction velocity in the median nerve (distal latency 5.9 ms, conduction velocity 33 m/s, amplitude 2.9 mV). EMG showed mild denervation activity and increased amplitude of the motor unit potentials. Overall, the results were consistent with combined demyelination (slowed conduction velocity and increased distal latency) and axonal damage (low neurographic amplitudes, denervation activity, and increased amplitude of the motor unit potentials). MRI showed cerebellar, pontine, and generalized cerebral atrophy (Fig. 1a, b)

Blood chemistry showed hypoalbuminemia ranging from 25 to 30 g/L (normal 39–47 g/L), and elevated total cholesterol 9.5 mmol/L (normal 3.3–7.7 mmol/L) and LDL 6.9 mmol/L (normal 1.8–5.7 mmol/L). AFP levels were within normal range.

**Fig. 2:**
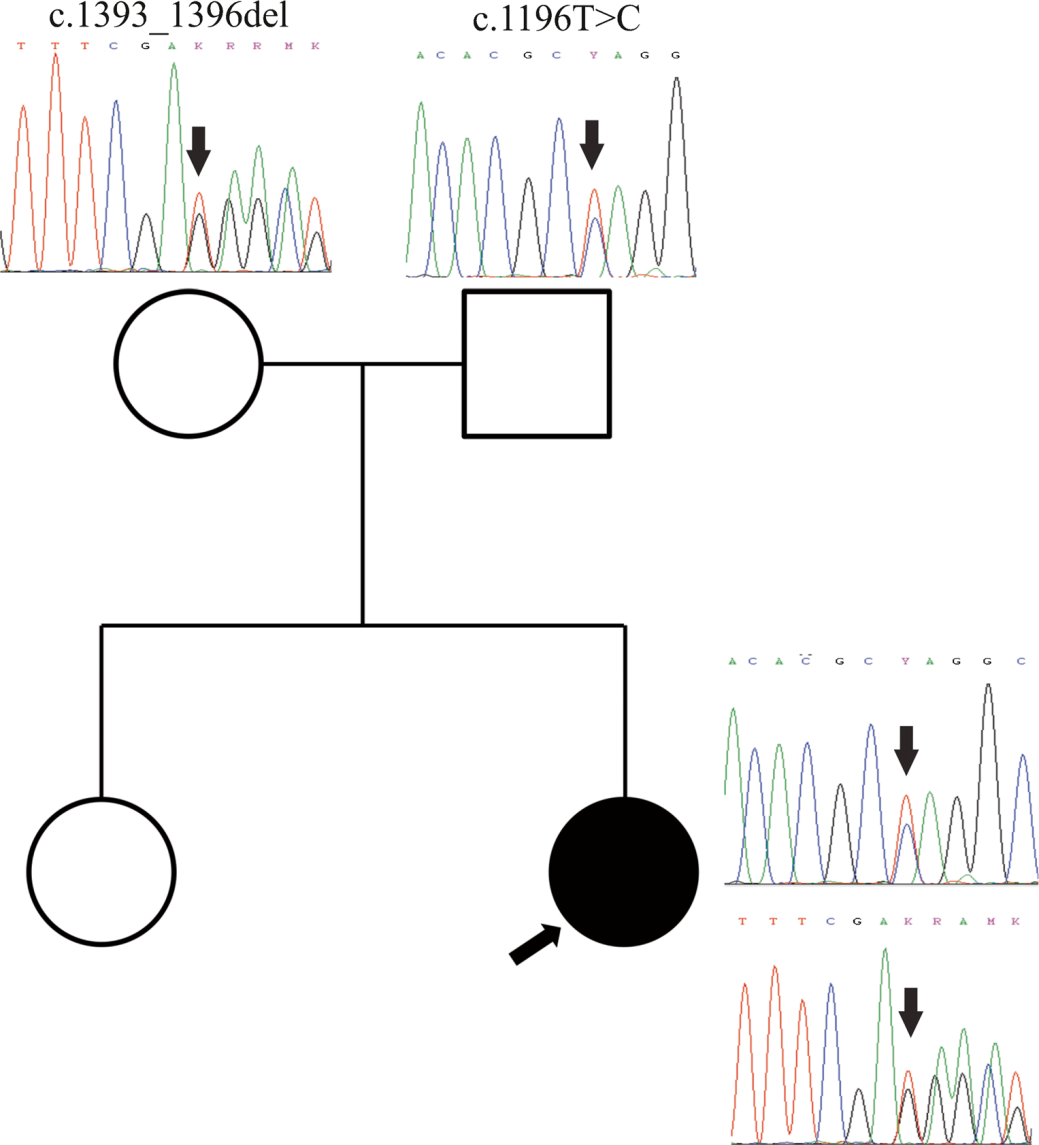
Genetic findings. Pedigree of the family showing the patient (*black circle* and *arrow*) and her healthy sister and parents. Chromatograms show the location of the mutations (*arrows*) in each individual

Her edema was treated with repeated albumin replacement therapy and compressive limb therapy (Lymphopress, I-Tech, Italy) resulting in some improvement (Figs. 1d, e and 2).

### Genetics

DNA was purified from blood using standard methods. Whole-exome sequencing was performed at HudsonAlpha Institute for Biotechnology (Huntsville, AL) using Roche-NimbleGen Sequence Capture EZ Exome v3 kit and paired-end 100 nt sequencing on the Illumina HiSeq. Read mapping, variant calling, and filtering was performed as described previously [5].

PCR-amplification and Sanger sequencing of the *PNKP* gene was performed by standard methods (primers and conditions available upon request). Biallelic mutations were found in 14 genes of which only *PNKP* was consistent with the patient’s phenotype (Table 1). The *PNKP* gene (NCBI reference sequence NM_007254) contained two heterozygous mutations, one in exon 14 (c.1196 T > C, p.Leu399Pro) and one in exon 16 (c.1393_1396del, p.Glu465*). Sanger sequencing confirmed the mutations and sequencing of the parents showed that they were located on different alleles. The c.1196 T > C was absent in 400 in-house controls and the 1000 genomes database and had an allele frequency of 6 × 10^−4^ (no homozygous individuals) at the Exome Aggregation Consortium (ExAC), Cambridge, MA (URL: http://exac.broadinstitute.org). It is predicted to change a highly conserved leucine for proline in position 399. The c.1393_1396del non-sense mutation has not been previously reported and was absent in our controls and public databases. It is predicted to introduce a stop codon and premature truncation of the protein. Based on their very low frequency, predicted biological impact and compatibility with the phenotype, the mutations were considered pathogenic.

**Table 1:** Major clinical features of the four AOA subtypes

Disease	Gene	Ataxia	OA	PN	Extrap*	Cogn	Alb	Chol	AFP
AOA1	*APTX*	+	+	+	+	+/−	+	+	−
AOA2	*SETX*	+	+	+	+	+/−	−	+	+
AOA3	*PIK3R5*	+	+	+	−	−	−	−	+
AOA4	*PNKP*	+	+	+	+	+	+	+	+

*OA* oculomotor apraxia, *PN* peripheral neuropathy, *Extrap* extrapyramidal (choreoathetosis, dystonia, tremor), *Cogn* cognitive decline, *Alb* hypoalbuminemia, *Chol* hypercholesterolemia, *AFP* elevated AFP

## Discussion

We report a sporadic case of AOA4 caused by known and novel mutations of the *PNKP* gene. This is only the ninth reported family and the first independent replication of *PNKP* mutations as a cause of AOA in a different population.

Our patient’s most striking clinical feature was the severe bilateral oedema of the lower limbs due to hypoalbuminemia. Limb edema affects approximately half the patients with AOA1, but has not been previously reported in AOA4 [4, 6]. Otherwise, the patient had typical clinical features for this disorder including peripheral neuropathy, cognitive impairment, and hypercholesterolemia, but no extrapyramidal dysfunction which was present in the majority of the reported patients (9/11) [4].

In conclusion, our findings confirm the pathogenic role of *PNKP* in an independent, novel case of AOA4. Moreover, we show that *PNKP* mutations should be considered in the differential diagnosis of recessive and/or sporadic ataxia also in non-Portuguese populations.

## References

[1] Coutinho P, Barbot C. Ataxia with Oculomotor Apraxia Type 1. In: Pagon RA, Adam MP, Ardinger HH, Wallace SE, Amemiya A, Bean LJH, Bird TD, Fong CT, Mefford HC, Smith RJH, Stephens K, editors. GeneReviews(R). Seattle (WA); 1993.

[2] Moreira MC, Koenig M. Ataxia with Oculomotor Apraxia Type 2. In: Pagon RA, Adam MP, Ardinger HH, Wallace SE, Amemiya A, Bean LJH, Bird TD, Fong CT, Mefford HC, Smith RJH, Stephens K, editors. GeneReviews(R). Seattle (WA); 1993.

[3] Al Tassan N, Khalil D, Shinwari J, Al Sharif L, Bavi P, Abduljaleel Z (2012). A missense mutation in PIK3R5 gene in a family with ataxia and oculomotor apraxia. Hum Mutat.

[4] Bras J, Alonso I, Barbot C, Costa MM, Darwent L, Orme T (2015). Mutations in PNKP cause recessive ataxia with oculomotor apraxia type 4. Am J Hum Genet.

[5] Haugarvoll K, Johansson S, Tzoulis C, Haukanes BI, Bredrup C, Neckelmann G (2013). MRI characterisation of adult onset alpha-methylacyl-coA racemase deficiency diagnosed by exome sequencing. Orphanet J Rare Dis.

[6] Le Ber I, Moreira MC, Rivaud-Pechoux S, Chamayou C, Ochsner F, Kuntzer T (2003). Cerebellar ataxia with oculomotor apraxia type 1: clinical and genetic studies. Brain.

